# Insulin Edema Associated With Glargine

**DOI:** 10.1210/jcemcr/luad158

**Published:** 2023-12-19

**Authors:** Jessica Wood, Varun Samji, Francesco S Celi, Priyanka Majety

**Affiliations:** Division of Endocrinology, Diabetes and Metabolism, Virginia Commonwealth University Health, Richmond, VA 23298, USA; Division of Endocrinology, Diabetes and Metabolism, Virginia Commonwealth University Health, Richmond, VA 23298, USA; Department of Medicine, University of Connecticut, UConn Health 263 Farmington Avenue, Farmington, CT 06030-8075, USA; Division of Endocrinology, Diabetes and Metabolism, Virginia Commonwealth University Health, Richmond, VA 23298, USA

**Keywords:** insulin, edema, glargine, type 2 diabetes mellitus

## Abstract

Insulin edema is a poorly understood complication of insulin therapy. It has been reported in patients with both type 1 and 2 diabetes mellitus and typically occurs in patients with newly diagnosed or poorly controlled diabetes mellitus either after initiation or intensification of insulin therapy. A 20-year-old man presented with anorexia, polydipsia, and weight loss. Serum glucose on admission was 824 mg/dL (45.8 mmol/L) and hemoglobin A1c was >14.0. Additional workup was notable for positive anti-IA2 antibodies and low C-peptide of 0.5 ng/mL (1.1-4.4 ng/mL). He was diagnosed with type 1 diabetes mellitus and was started on insulin therapy with glargine and lispro. Within 4 days after insulin initiation, he developed bilateral leg swelling and reported a 25-pound (11.3-kg) weight gain over the next 10 days. After excluding other systemic causes of edema such as heart failure, renal failure, and liver failure, a diagnosis of insulin edema was made. Insulin glargine was switched to insulin degludec. Complete resolution of edema occurred within 3 days of switching the insulins. Insulin edema is a diagnosis of exclusion. Insulin's role in renal sodium handling, vasodilation, and increased vascular permeability have been postulated as possible mechanisms. Clinicians should be aware of this rare complication.

## Introduction

Insulin edema is an uncommon and poorly understood complication of insulin therapy occurring in patients with diabetes. It has been reported in patients with both type 1 diabetes mellitus (T1DM) and type 2 diabetes mellitus (T2DM) and typically occurs in patients with newly diagnosed or poorly controlled diabetes mellitus either after initiation or intensification of insulin therapy. Although the condition is usually self-limiting, progression to overt cardiac failure and development of pleural effusion have been reported [1].

The pathophysiology of insulin edema is not well understood. It likely involves the interaction of several factors including the insulin analog used, vasodilation, renal sodium handling, and counterregulatory hormones, such as glucagon, cortisol, and catecholamines, and alterations in properties of subcutaneous tissue [2, 3].

We report a patient with T1DM who developed edema within days of initiating insulin glargine that resolved with discontinuation of that medication. There have been few case reports in the literature of insulin edema with glargine, which are reviewed here. It is important to be aware of this rare complication, and its occurrence should be documented and differentiated from other causes of edema.

## Case Presentation

A 20-year-old African American man with no significant medical history presented to the emergency department with a 1-month history of abdominal pain, nausea, vomiting, polydipsia, and weight loss totaling 15 pounds. He was on no medications or supplements. His family history was significant for a paternal aunt with diabetes (unknown type).

## Diagnostic Assessment

On physical examination, he appeared to be in mild distress resulting from nausea but otherwise had a normal examination. Laboratory data obtained on admission were notable for hemoglobin A1c >14.0%, serum glucose of 824 mg/dL (45.8 mmol/L), and a trace of urine ketones with a normal arterial blood pH of 7.39 (7.35-7.45). His weight on admission was 58.6 kg (128.9 lb). Additional workup was notable for negative antiglutamic acid decarboxylase and insulin antibodies but positive anti-IA2 (insulinoma-associated protein-2) antibodies.

## Treatment

He was started on weight-based insulin therapy with basal insulin glargine 10 U daily and prandial lispro 3 U with meals. Blood glucose levels improved before discharge.

## Outcome and Follow-up

He was closely monitored over the next several days via phone calls by our diabetes care team. He was noted to have persistent fasting and postprandial hyperglycemia, and his insulin doses were progressively increased. Three days after discharge, he noted the onset of bilateral leg swelling and weight gain that was progressive.

**Figure 1.**
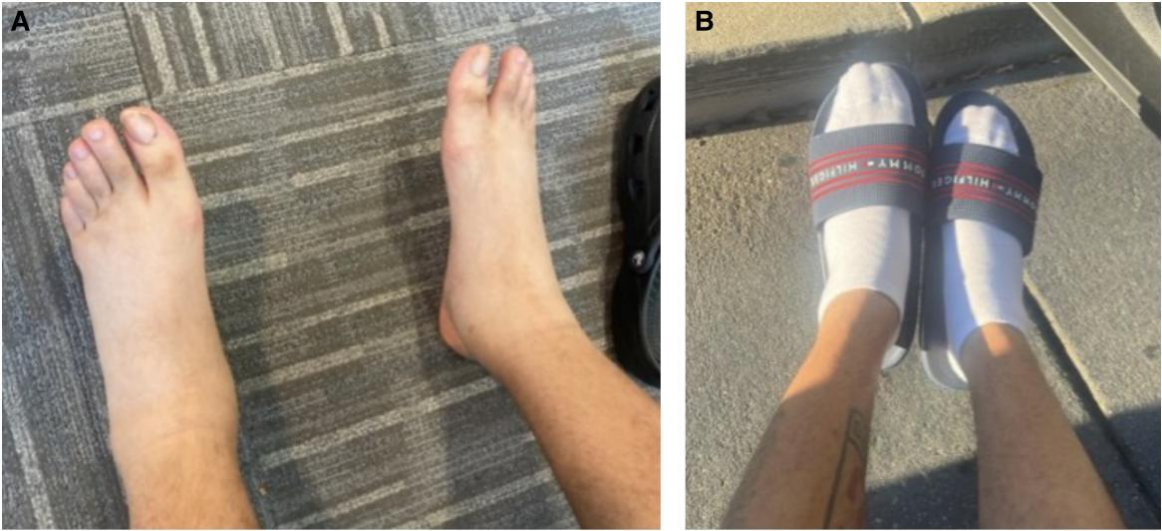
Bilateral pedal edema that resolved with discontinuing insulin glargine. (A) Pitting edema in bilateral feet extending up to the patient’s calves during clinic visit, 10 days after initiating insulin glargine. (B) Resolution of edema on day 3 after discontinuing insulin glargine.

Ten days after discharge, he was seen in the clinic for further evaluation. The patient denied any shortness of breath, cough, chest pain, orthopnea, or nocturnal dyspnea. He was found to have extensive 2+ pitting edema in bilateral feet extending up to his calves (Fig. 1A). Dorsalis pedis pulses were 2+ bilaterally. No warmth or erythema of the legs was noted. His weight in the clinic was 70.2 kg (154.4 lb), up 11.6 kg (25.5 lb). Insulin edema was suspected because of the clinical presentation, his young age, and lack of suspicion for other etiologies. Because this is a diagnosis of exclusion, a workup to exclude other causes of edema was performed, as outlined in Table 1.

**Table 1.** Laboratory tests obtained during evaluation

Laboratory values	Results	Reference range
Hemoglobin A1C	>14%	<5.7%
Anti-GAD	<5.0 U/mL	<5.0 U/mL
Insulin antibodies	<5.0 uU/mL	<5.0 uU/mL
Zinc transporter 8 antibodies	<15 U/mL	<15 U/mL
IA-2 autoantibodies	8.9 U/mL	<7.5 U/mL
C-peptide	0.5 ng/mL Serum glucose: 599 mg/dL (33.2 mmol/L)	1.1-4.4 ng/mL
eGFR	>120 mL/min/1.73 m^2^	≥60 mL/min/1.73 m^2^
Creatinine	0.54 mg/dL	0.60-1.20 mg/dL
Albumin/creatinine ratio, urine	<30 mg/g Crea	0-30 mg/g Crea
Aspartate aminotransferase	16 U/L	0-50 U/L
Alanine aminotransferase	18 U/dL	0-60 U/L
Brain natriuretic peptide	11 pg/mL	≤100 pg/mL
TSH	1.40 uIU/mL	0.35-4.94 uIU/mL
T4, free, direct	0.9 ng/dL	0.7-1.5 ng/dL
Albumin	3.6 g/dL	3.7-5.2 g/dL
Antinuclear antibody	Negative at 1:80	Negative at 1:80

Abbreviations: BG, blood glucose; eGFR, estimated glomerular filtration rate; IA-2, insulinoma-associated protein-2.

Laboratory data were notable for normal liver enzymes, creatinine, glomerular filtration rate, and urine albumin levels, ruling out hepatic and renal causes of edema. Thyroid function tests were normal and antinuclear antibodies were negative. His presentation was discussed with cardiology and brain natriuretic peptide level was drawn, which was also normal. No further cardiac workup was recommended at that time given the patient's history and lack of other clinical signs or symptoms of heart failure. After ruling out systemic causes of edema in our patient, a diagnosis of insulin edema was made. Based on prior case reports of glargine-associated insulin edema, he was switched from insulin glargine to insulin degludec. He noted significant improvement within 24 hours of switching insulin with a 6.4-lb (2.9 kg) weight loss in just 1 day and complete resolution of edema within 3 days (Fig. 1B). He had no recurrence of edema once glargine was discontinued despite increasing insulin dosing for glycemic control.

## Discussion

Several side effects have been reported during the treatment with insulin glargine, such as hypoglycemia and injection site reactions. Rarely, insulin glargine may induce edema. Insulin edema can occur in patients with poorly controlled T1DM or T2DM, typically after initiation or intensification of insulin therapy, and is a diagnosis of exclusion. It was first described in 1928 and only a few cases have been reported since. Its true incidence is unknown but is estimated to be approximately 3.5% [2]. Although the presentation is usually acute and self-limiting dependent edema, reports of cardiopulmonary congestion or ascites and recurrence have been published [1].

The pathophysiology of insulin edema is unclear and various mechanisms have been proposed [2]. Insulin promotes renal resorption of sodium by stimulating Na+/K+ ATPase in distal tubules [2]. It causes vasodilation possibly via stimulation of Na, K-ATPase, β-adrenergic stimulation, stimulation of calcium ion-activated potassium ion channels, and stimulation of nitric oxide release [2]. Increased vascular permeability secondary to expression of vascular endothelial growth factors and alterations in the properties of endothelial cells resulting from insulin has also been postulated. In addition, in patients with poor glycemic control, persistent hyperglycemia also contributes to transcapillary leakage of albumin [4].

Risk factors for developing insulin edema include T1DM, high A1C, high dose of insulin, rapid correction of hyperglycemia, presentation in ketoacidosis, and poor nutritional status [2]. Additional risk factors for developing insulin edema include thiamine deficiency and an underlying genetic predisposition related to a mitochondrial mutation, although this seems to be rare [2]. It is still unclear why insulin edema is present only in certain patients.

Reports of insulin edema occurring with various analogs alone or in combination have been published. However, its occurrence with insulin glargine may have additional pathogenic hypothesis. Insulin glargine is a first generation long-acting insulin analog that precipitates at physiologic pH in subcutaneous tissue after injection, delaying the absorption. It has been hypothesized that these microprecipitates of insulin glargine could lead to alterations in subcutaneous tissue that can impede blood and lymphatic flow, leading to swelling. Switching insulin glargine to insulin detemir has shown resolution of edema in some case reports [3]. We switched our patient from insulin glargine to insulin degludec, which is a second-generation long-acting insulin analog with a fatty acid side chain. Insulin degludec forms multihexamers on administration into subcutaneous tissue, resulting in a soluble depot, from which it is slowly absorbed, in contrast to insulin glargine, which forms microprecipitates. In a 5-year study, peripheral edema was reported in 20% of patients with T2DM with glargine use [5]. With insulin degludec, edema occurred in 0.9% and 3% of patients with T1DM and T2DM, respectively [6].

Including the current case, 5 cases of glargine-associated insulin edema in adults have been reported so far, as outlined in Table 2. Both patients with T1DM (3) and T2DM (2) were affected. Time to onset of edema after glargine initiation varied between 4 and 14 days. Edema resolved with discontinuation of glargine, switching to other analogs, sodium restriction, diuretics or spontaneously. Time to resolution was between 3 days and 1 week.

**Table 2.** Review of literature on edema with insulin glargine in adults

No.	Case reports	Age/sex	Classification of diabetes; Duration	A1C at diagnosis	Onset (days)	Antihyperglycemic regimen	Treatment of edema	Time to resolution of edema	Comments
1	Adamo et al [7]	20/F	T1DM, 18 y	18.8%	Within 6 d	Glargine Lispro	IV Furosemide Insulin dose reduction	5 d	Generalized edema
2	Elmahal et al [8]	35/F	T2DM; 8 y	12.3%	Within 14 d; “almost immediate”	Glargine Metformin Gliclazide Sitagliptin	Sodium restriction and IV furosemide	1 wk	Generalized edema
3	Wong et al [9]	40/F	T1DM; ND	13.1%	Within 4 d	Glargine Glulisine	Spironolactone	Within 1 week	Generalized edema
4	Succurro et al [3]	72/M	T2DM; 15 y	12.3%	Within 14 d	Glargine alone. Previously on metformin	Switched from glargine to insulin detemir	Within a “few days”	Wood, firm non-pitting edema
5	Current case	20/M	T1DM; ND	>14%	Within 4 days	Glargine Lispro	Switched from glargine to insulin degludec	Within 3 days	Bilateral pitting pedal edema

Abbreviations: ND, newly diagnosed; T1DM, type 1 diabetes mellitus; T2DM, type 2 diabetes mellitus.

Here, we report a patient with a new diagnosis of T1DM who developed edema after initiating insulin therapy, and whose edema subsided after switching to another basal insulin analog. Insulin glargine is the most likely culprit in our patient. The Naranjo scale showing probable association (score 7) further supports our hypothesis [10]. Insulin degludec and insulin detemir have a fatty acid side chain and do not form microprecipitates after injection [3, 6]. Previous case reports demonstrated resolution of edema after switching insulin glargine to insulin determir [3]. This is the first case report that demonstrates that insulin degludec can also be considered. Because the edema resolved promptly with switching insulin analogs, we did not rechallenge with glargine. We conclude that in patients who develop edema with insulin glargine, it is worthwhile to consider switching to different basal insulin analog that does not form microprecipitates, such as degludec or detemir.

## Learning Points

- Insulin edema is a rare and poorly understood complication of insulin therapy occurring in patients with both type 1 and type 2 diabetes mellitus.
- Several factors including the insulin analog used, vasodilation, vascular permeability, renal sodium handling, counterregulatory hormones, and alterations in properties of subcutaneous tissue are thought to be involved in its pathogenesis.
- Insulin edema is a diagnosis of exclusion. Clinicians should be aware of this rare complication, and its occurrence should be documented and differentiated from other causes of edema.

## Data Availability

Original data generated and analyzed during this study are included in this published article.

## Abbreviations

T1DM: type 1 diabetes mellitus
T2DM: type 2 diabetes mellitus
